# Effect of type 1 diabetes on the production and vasoactivity of hydrogen sulfide in rat middle cerebral arteries

**DOI:** 10.1002/phy2.111

**Published:** 2013-10-20

**Authors:** Elosie Y Streeter, Emilio Badoer, Owen L Woodman, Joanne L Hart

**Affiliations:** School of Medical Sciences and Health Innovations Research Institute, RMIT UniversityBundoora, Victoria, 3083, Australia

**Keywords:** Cerebral artery, diabetes, hydrogen sulfide, oxidative stress

## Abstract

Hydrogen sulfide (H_2_S) is produced endogenously in vascular tissue and has both vasoregulation and antioxidant effects. This study examines the effect of diabetes-induced oxidative stress on H_2_S production and function in rat middle cerebral arteries. Diabetes was induced in rats with streptozotocin (50 mg/kg, i.v.). Middle cerebral artery function was examined using a small vessel myograph and superoxide anion generation measured using nicotinamide adenine dinucleotide phosphate (NADPH)-dependent lucigenin-enhanced chemiluminescence. Cystathionine-γ-lyase (CSE) mRNA expression was measured via RT-PCR. Diabetic rats had elevated blood glucose and significantly reduced cerebral artery endothelial function. Maximum vasorelaxation to the H_2_S donor NaHS was unaffected in diabetic cerebral arteries and was elicited via a combination of K^+^, Cl^−^, and Ca^2+^ channel modulation, although the contribution of Cl^−^ channels was significantly less in the diabetic cerebral arteries. Vasorelaxation to the H_2_S precursor l-cysteine and CSE mRNA were significantly increased in diabetic cerebral arteries. Cerebral artery superoxide production was significantly increased in diabetes, but this increase was attenuated ex vivo by incubation with the H_2_S donor NaHS. These data confirm that cerebral artery endothelial dysfunction and oxidative stress occurs in diabetes. Endogenous H_2_S production and activity is upregulated in cerebral arteries in this model of diabetes. Vasorelaxation responses to exogenous H_2_S are preserved and exogenous H_2_S attenuates the enhanced cerebral artery generated superoxide observed in the diabetic group. These data suggest that upregulation of endogenous H_2_S in diabetes may play an antioxidant and vasoprotective role.

## Introduction

Hydrogen sulfide (H_2_S) is an endogenous mediator with important vascular effects, including vasorelaxation and vascular protection (Streeter et al. 2013). In the vasculature H_2_S is produced by cystathionine-γ-lyase (CSE) and the combination of 3-mercaptopyruvate sulfurtransferase and cysteine aminotransferase (Shibuya et al. 2009). H_2_S is well established as a vasodilator in the periphery and there are now several lines of evidence that it also dilates cerebral vessels (Leffler et al. 2011; Streeter et al. 2012). CSE has been shown to be present in pig pial arteries (Leffler et al. 2011) and rat middle cerebral arteries (MCA) (Streeter et al. 2012). The CSE substrate l-cysteine causes CSE-dependent vasorelaxation in cerebral vessels (Leffler et al. 2011; Streeter et al. 2012) suggesting a physiological role for endogenous H_2_S. Acute, exogenous H_2_S causes cerebral vasodilation via mechanisms involving hyperpolarization and ion channels including K_ATP_ channels (Leffler et al. 2011; Liang et al. 2011), Ca^2+^ channels (Streeter et al. 2012; Tian et al. 2012), and Cl^−^ channels (Streeter et al. 2012).

H_2_S is a potent one-electron chemical reductant that is theoretically capable of scavenging free radicals by single electron or hydrogen atom transfer (Carballal et al. 2011). Thus, H_2_S may participate in many reactions (Stasko et al. 2009) and is reported to scavenge reactive oxygen species (ROS) and reactive oxygen nitrogen species, however, the kinetics, reactivity, and mechanism of H_2_S interactions with ROS are poorly understood under physiological conditions (Carballal et al. 2011). H_2_S at nanomolar concentrations has been reported to inhibit superoxide formation in both endothelial (Muzaffar et al. 2008a) and vascular smooth muscle cells (Muzaffar et al. 2008b) by reducing nicotinamide adenine dinucleotide phosphate (NADPH) oxidase expression and activity.

Diabetes causes peripheral and cerebrovascular disease, the hallmarks of which are endothelial dysfunction and atherosclerosis. Diabetic cerebrovascular disease increases the risk of ischemic stroke by twofold (Quinn et al. 2011) and this increased risk has been associated with diabetic cerebrovascular disease (Nazir et al. 2006; Gunarathne et al. 2009). In diabetes, hyperglycemia and oxidant stress precede endothelial dysfunction. It is well established that vascular superoxide generation is increased in large and small vessels in diabetes (Leo et al. 2010, 2011a,c2011c), accompanied by eNOS uncoupling and increased NOX2 expression (Leo et al. 2011a). The effect of the increased oxidative stress is a reduction in endothelium-dependent NO signaling and impaired endothelium-derived hyperpolarizing factor (EDHF)-mediated vasorelaxation (Leo et al. 2011c). Thus, the mechanism of endothelium-dependent vasorelaxation is altered by diabetes-induced oxidative stress.

There is also evidence that H_2_S production and vasodilator capacity are altered in diabetic peripheral vessels. In aorta, mesenteric and pulmonary arteries, type 1 diabetes has been shown to enhance the vasodilator actions of H_2_S (Denizalti et al. 2011). Aorta from nonobese diabetic mice have enhanced vasodilation by H_2_S, as well as increased CSE mRNA expression (Brancaleone et al. 2008).

In cerebral vessels, the effect of diabetes on the vasodilator response or production of H_2_S has not been studied. To further the understanding of cerebrovascular pathological changes induced by diabetes, this study investigated whether diabetes alters the middle cerebral artery production or vascular response to H_2_S.

## Methods

### Ethical approval

All procedures were performed to conform to the guidelines set out by the National Health and Medical Research Council of Australia and were approved by the RMIT University Animal Ethics Committee.

### Induction of diabetes

Male Sprague Dawley rats were obtained from the Animal Resources Centre (ARC, Canning Vale, Western Australia). The animals were housed in a temperature-controlled room on a 12:12 h light/dark cycle in the RMIT Animal Facility (RMIT Bundoora West campus, Victoria, Australia). Type 1 diabetes was induced in rats as previously described (Leo et al. 2011c). Briefly, male Sprague Dawley rats 8 weeks old (Animal Resource Centre, Perth WA, Australia) were administered a single injection of streptozotocin (STZ) (50 mg/kg) in citrate buffer via tail vein injection after the rats were fasted overnight. Diabetic animals were housed until 16 weeks of age to allow for development of vascular disease. Animals for the control group were obtained at 15 weeks and housed until 16 weeks before use. Following this, rats were asphyxiated by CO_2_ inhalation, followed by decapitation, at which point blood was collected and blood glucose levels measured using an Accu-Check Performa® (Roche, Germany) blood glucose monitor. Induction of diabetes was considered successful by a nonfasting blood glucose concentration of >25 mmol/L.

### Isolated cerebral artery preparation

Rat brains were collected into ice-cold Krebs’ solution (composition [mmol/L]: NaCl, 119; KCl, 4.7; MgSO_4_ 1.2; CaCl_2_, 2.5; KH_2_PO_4_, 1.2; NaHCO_3_, 25; Glucose 11.1; ethylenediaminetetraacetic acid (EDTA) 0.26, pH 7.4 and gassed with 95% O_2_, 5% CO_2_). Proximal lengths of the MCA (∼250 μm in diameter) were dissected and cut into 2 mm segments before being mounted into a 610M 4-chamber wire myograph (Danish Myotechnology, DMT, Aarhus, Denmark), filled with oxygenated Krebs’ solution, and warmed to 37°C. Changes in isometric tension were recorded via Myodaq software (DMT, Aarhus, Denmark).

### Experimental protocol

Due to the high level of spontaneous myogenic tone in rat MCA, the following protocol was adopted to standardize the level of passive force applied to each vessel (Favaloro et al. 2003; Streeter et al. 2012). First, a 4 mN force was applied to each segment over a 30-min period to allow spontaneous tone to develop. Subsequently, Krebs’ solution was replaced with a calcium free Krebs’ solution (composition as above, excluding the CaCl_2_, and replacing EDTA with ethylene glycol tetraacetic acid (EGTA) 2 mmol/L), causing the vessels to fully relax. The passive force was reset to 4mN, before reintroducing normal Krebs’ solution to allow spontaneous redevelopment of tone. The viability of the vascular smooth muscle was confirmed by the development of spontaneous tone. Inclusion criteria required the development of at least 2.5mN of spontaneous tone. Bradykinin (100 nmol/L) was then applied to assess the viability of the endothelium.

### Vasorelaxation to exogenous and endogenous H_2_S

The relaxation response of vessel segments from diabetic and control rats to cumulative concentrations of each of the following drugs was assessed in separate vessel segments: NaHS (H_2_S donor, 10μmol/L–3 mmol/L) in the presence and absence of KCl (50 mmol/L) to block K^+^ conductance; 4,4′-diisothiocyanatostilbene-2,2′-disulfonic acid (DIDS, 300 μmol/L), an anion transport inhibitor that inhibits Cl^−^ uptake; nifedipine (10 μmol/L), a l-type Ca^2+^ channel inhibitor. Concentration–response curves to l-cysteine (10 μmol/L-100 mmol/L), the precursor to endogenous H_2_S formation, were conducted in the presence or absence of the CSE inhibitor, propargylglycine (PPG, 20 mmol/L).

### Vascular superoxide assay

Segments of cerebral arteries from the Circle of Willis and basilar artery were dissected and pooled and superoxide production measured by lucigenin-enhanced chemiluminescence (Miller et al. 2005). Cerebral artery segments were preincubated for 45 min at 37°C in Krebs-HEPES buffer (composition [mmol/L]: NaCl 99.9, KCl 4.7, KH_2_PO_4_ 1.0, MgSO_4_.7H_2_O 1.2, D-glucose 11.0, NaHCO_3_ 25.0, CaCl_2_.2H_2_O 2.5, Na HEPES 20.0, pH 7.4) containing diethylthiocarbamic acid (1 mmol/L) to inactivate superoxide dismutase, and NADPH (100 μmol/L) as a substrate for NADPH oxidase. Diphenylene iodonium (DPI, 1 μmol/L) was used to inhibit NADPH oxidase in some wells. Krebs-HEPES buffer (300 μL) containing lucigenin (5 μmol/L) was placed in a 96-well optiplate which was loaded into a Polarstar Optima photon counter (BMG Labtech, Melbourne, VIC, Australia) to measure background photon emission at 37°C. After background counting was completed, cerebral artery segments were added to each well and photon emission was recounted. The background reading was subtracted from the superoxide anion counts and normalized to dry tissue weight, and superoxide levels expressed as 10^4^ counts sec^−1^ mg^−1^.

### Real time PCR

#### RNA extraction and quantification

Middle cerebral arteries were collected and stored in RNAlater® solution (Invitrogen, Melbourne, Australia) at −20°C. For RNA extraction, MCA were removed from RNAlater® solution and placed in 1 mL of TRIzol® (Invitrogen) and homogenized, then 200 μL chloroform was added and the sample was vigorously shaken. Samples were allowed to stand for 5 min and spun at 12,000 g for 15 min at 4°C after which the upper aqueous phase was transferred to a new tube. The aqueous phase was precipitated by mixing with 500 μL ice-cold isopropanol alcohol. Samples were incubated at −20°C for 1 h and then centrifuged at 13,200 g for 15 min at room temperature. The supernatant was removed and the resulting pellet was washed with 200 μL of 75% ethanol in RNase-free water. After centrifugation at 13,200 g for 10 min at room temperature, the ethanol supernatant was removed and the RNA pellets were left to air dry for 5–10 min before being redissolved in 40 μL of RNase-free water by mixing and then incubating at 55°C for 10 min. The quality and quantity of extracted RNA was determined on a NanoDrop 1000 spectrophotometer (Nanodrop Technologies, Wilmington, DE) by measuring absorbance at 260 nm and 280 nm with a 260/280 ratio of ∼1.7 recorded for all samples. The RNA samples were diluted as appropriate to equalize concentrations, and stored at −80°C for subsequent reverse transcription.

#### Reverse transcription and real-time PCR

First-strand complementary DNA (cDNA) synthesis was performed using commercially available TaqMan Reverse Transcription Reagents (Invitrogen) in a final reaction volume of 20 μL. A negative sample containing a randomly chosen sample with no Reverse Transcriptase (Superscript®, Invitrogen, Melbourne, Australia) was prepared to demonstrate an absence of PCR products in amplifications of cDNA during the real-time PCR cycling. A serially diluted pooled RNA sample from the control group was produced and also included to ensure efficiency of reverse transcription and for calculation of a standard curve for real-time quantitative polymerase chain reaction (RT-PCR). All RNA, negative control, and standard samples were reverse transcribed to cDNA in a single run from the same reverse transcription master mix. Quantification of mRNA (in duplicate) was performed on a 72-well Rotor-Gene 3000 Centrifugal Real-Time Cycler (Corbett Research, Mortlake, Australia). Taqman-FAM-labeled primer/probe for cystathionine gamma-lyase (Cat No. Rn00567128_m1) was used in a final reaction volume of 20 μL. PCR conditions were 2 min at 50°C for Uracil-N-glycosylase activation, 10 min at 95°C then 40 cycles of 95°C for 15 sec, and 60°C for 60 sec. 18S ribosomal RNA (18S rRNA) (Cat No. Hs99999901_s1) was used as a housekeeping gene to normalize threshold cycle (CT) values. The relative amounts of mRNAs were calculated using the relative quantification (ΔΔCT) method (Livak and Schmittgen 2001).

### Data analysis and statistics

Results are expressed as mean ± standard error of the mean with the number of experiments denoted by n. Concentration–response curves to NaHS and l-cysteine are expressed as a percentage reversal of the spontaneous contraction. These data were computer fitted to a sigmoidal curve using nonlinear regression (Graphpad Prism, version 5.0, Graphpad Software Incorporated, La Jolla, CA) to calculate the sensitivity of the vasorelaxation response (pEC_50_ =−log[concentration eliciting 50% of the maximal response]). Fold shift was calculated by dividing the EC_50_ in the presence of the inhibitor and by the EC_50_ in the absence of the inhibitor. Statistical analysis was performed using either unpaired t-tests or by one-way analysis of variance (ANOVA) with post hoc tests applied as indicated (GraphPad Prism, Version 5, Graphpad Software Incorporated). *P* < 0.05 was considered statistically significant.

### Drugs and reagents

All drugs and reagents were purchased from Sigma-Aldrich (St Louis, Missouri). All drugs were dissolved in distilled water.

## Results

### Induction of type 1 diabetes

Diabetic rats had significantly higher blood glucose than controls (blood glucose [mmol/L]: Control 8.7 ± 0.5, Diabetic 30.9 ± 0.8, *n* = 13–17, *P* < 0.0001), confirming induction of diabetes.

### Endothelial dysfunction in type 1 diabetic MCA

The level of spontaneous tone developed in middle cerebral artery segments was not significantly different between vessels from control and diabetic animals (Control: 7.7 ± 0.5 mN; Diabetic: 8.0 ± 0.3 mN, *n* = 9) nor was the maximal contractile capacity (to 125 mmol/L KCl) significantly different in cerebral artery from control and diabetic animals (Control: 8.7 ± 0.4 mN, Diabetic: 8.9 ± 0.6 mN, *n* = 9), indicating no change in vascular smooth muscle function due to diabetes. However, relaxation of cerebral artery to BK 100 nmol/L was significantly reduced in diabetic animals, indicating endothelial dysfunction (Control: 77 ± 10, Diabetic: 43 ± 10, *n* = 5–6, *P* < 0.05).

### Effect of type 1 diabetes on vasorelaxation responses to exogenous H_2_S in MCA

**Figure 1:**
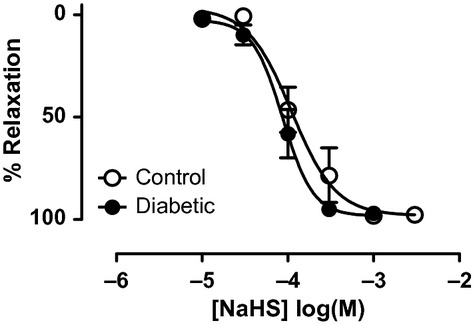
Vasorelaxation response to NaHS in control (○) and diabetic (•) cerebral arteries as a percentage reversal of the spontaneously developed tone. *n* = 7–9.

The H_2_S donor, NaHS, (10 μmol/L–3 mmol/L) produced a full, concentration-dependent vasorelaxation of cerebral artery which was not altered by diabetes (Fig 1, Table 1).

**Table 1:** Concentration–response curve parameters for NaHS in control and diabetic cerebral arteries

Group	Treatment	pEC_50_	Fold shift	Max relaxation (%)	*n*
Control	Untreated	3.99 ± 0.03		97 ± 3	7
	K^+^	3.55 ± 0.04^##^	2.7	99 ± 5	7
	DIDS	3.24 ± 0.05^###^	5.5	85 ± 4	7
	Nif	3.52 ± 0.11^##^	3.0	70 ± 7*	5
Diabetic	Untreated	4.08 ± 0.04		99 ± 4	9
	K^+^	3.72 ± 0.02^#^	2.3	90 ± 2	5
	DIDS	3.74 ± 0.07^#^	2.2Ψ	98 ± 6	5
	Nif	3.60 ± 0.05^###^	2.9	59 ± 3**	9

*P < 0.05, **P < 0.01 maximum relaxation versus untreated.

^#^*P* < 0.05, ^##^*P* < 0.01, ^###^*P* < 0.001 EC_50_ versus untreated for the respective group (control or diabetic).

Ψ Indicates less fold shift than control group for this treatment. K^+^, potassium chloride (50 mmol/L), DIDS, 4,4-diisothiocyanatostilbene-2,2-disulfonic acid (300 μmol/L); Nif, nifedipine (3 μmol/L).

### Effect of type 1 diabetes on mechanisms of H_2_S-induced vasorelaxation of MCA

**Figure 2:**
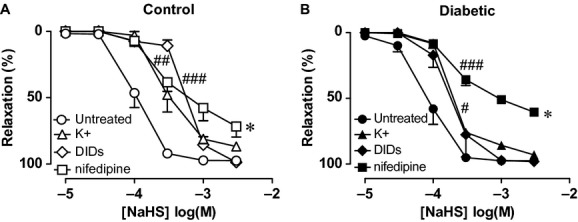
Vasorelaxation response to NaHS in (A). control and (B) diabetic cerebral arteries as a percentage of the spontaneously developed tone, in the presence and absence of KCl (50 mmol/L, triangles) to inhibit K^+^ conductance, the Cl^−^ conductance blocker DIDS (300 μmol/L, diamonds), and the l-type Ca^2+^ channel blocker nifedipine (10 μmol/L, squares). ^#^*P* < 0.05, ^##^*P* < 0.01, ^###^*P* < 0.001 for EC_50_ value versus control, *P < 0.05 maximum relaxation response versus control, analysis of variance, post hoc Dunnett's test, *n* = 5–9.

The contribution of K^+^ conductance, Cl^−^ conductance, and l-type Ca^2+^ channels to H_2_S-induced vasorelaxation in diabetic cerebral artery was investigated. Application of DIDS (300 μmol/L), an inhibitor of Cl^−^ conductance, produced a significant rightward shift of the NaHS concentration–response curve (Fig 2, Table 1). Reduction of K^+^ conductance using 50 mmol/L KCl significantly decreased the pEC_50_ of the NaHS-induced vasorelaxation (Fig 2, Table 1). Nifedipine (3 μmol/L) significantly attenuated the maximum relaxation and pEC_50_ to NaHS in cerebral artery (Fig 2, Table 1).

### Effect of type 1 diabetes on the vasorelaxation response to endogenous H_2_S

**Figure 3:**
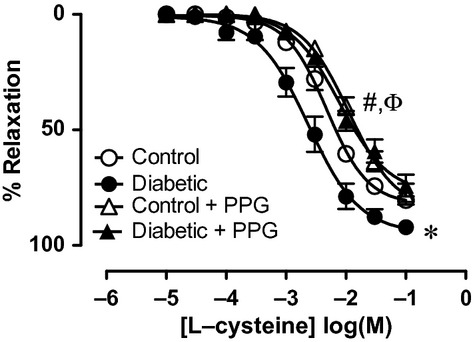
Vasorelaxation response to the CSE substrate l-cysteine in control (circles) and diabetic (triangles) cerebral arteries in the absence (open symbols) and presence (closed symbols) of the CSE inhibitor PPG (20 mM). ^#^*P* < 0.05 for EC_50_ value versus control, ^Φ^P<0.05 for EC_50_ values control and diabetic versus respective PPG treated, *P < 0.05 maximum relaxation response diabetic versus control, analysis of variance, post hoc Dunnett's test, *n* = 6–8.

The precursor for endogenous H_2_S formation, l-cysteine (10 μmol/L-100 mmol/L), caused concentration-dependent vasorelaxation of control cerebral arteries which was significantly enhanced by diabetes (Fig 3, Table 2). The CSE inhibitor, PPG (20 mmol/L) attenuated vasorelaxation to l-cysteine in control and diabetic cerebral artery (Fig 3, Table 2), indicating that the enhanced l-cysteine-induced vasorelaxation observed in diabetic cerebral artery was due to enhanced conversion of l-cysteine to H_2_S via CSE (Fig 3, Table 2).

**Table 2:** Concentration–response curve parameters for l-cysteine in control and diabetic cerebral arteries

Group	Treatment	pEC_50_	Fold shift	Max relaxation (%)	*n*
Control	Untreated	2.31 ± 0.05		84 ± 3	8
	PPG	1.94 ± 0.07^Ψ^	2.3	85 ± 5	7
Diabetic	Untreated	2.56 ± 0.09#		95 ± 5**	7
	PPG	2.10 ± 0.08^ΨΨ^	2.9	77 ± 5	8

** *P* < 0.01, maximum relaxation versus untreated control.

# *P* < 0.05, EC_50_, control versus diabetic.

^Ψ^*P*<0.05, ^ΨΨ^*P*<0.01, untreated versus PPG (control and diabetic).

### Effect of type 1 diabetes on the ability of tissues to produce hydrogen sulfide

**Figure 4:**
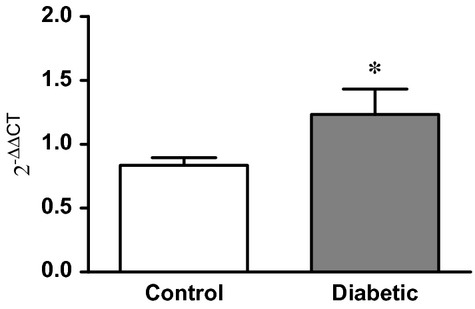
CSE mRNA expression in control (open bar) and diabetic (shaded bar) cerebral arteries, normalized to 18 sec rRNA expression. *P < 0.05, unpaired *t*-test, *n* = 3–7.

The mRNA expression of the H_2_S producing enzyme, CSE, as detected by real-time PCR, was significantly increased by 46% in diabetic compared to control cerebral artery (*P* < 0.05, Fig 4).

### Effect of exogenous hydrogen sulfide on vascular superoxide production

**Figure 5:**
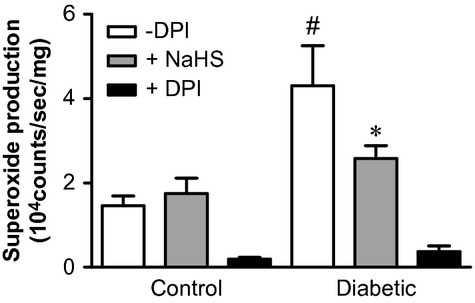
NADPH-stimulated superoxide production from control and diabetic cerebral vessels (Basilar artery pooled with the Circle of Willis arteries) in the absence and presence of the flavoprotein inhibitor DPI (5 μmol/L, to inhibit NADPH oxidase) or NaHS. ^#^*P* < 0.05 versus control, *P < 0.05 versus control treated with NaHS, paired *t*-test, *n* = 6.

For superoxide anion detection in cerebral arteries, the basilar artery was pooled with Circle of Willis cerebral arteries. Type 1 diabetes significantly enhanced NADPH-stimulated superoxide production in cerebral arteries (Fig 5). DPI (5 μmol/L), a flavoprotein inhibitor which inhibits NADPH oxidase (Selemidis et al. 2008), almost abolished superoxide production in cerebral arteries from both diabetic and control animals (Fig 5). Incubation of cerebral artery in NaHS (100 μmol/L), significantly reduced superoxide production in diabetic but not control cerebral artery (Fig 5).

## Discussion

This is the first study to examine the effect of type 1 diabetes on the response of cerebral arteries to exogenous and endogenous H_2_S. As expected, the results confirm the well-known fact that type 1 diabetes induces endothelial dysfunction and oxidative stress. While the maximal vasorelaxation response of cerebral arteries to exogenous H_2_S is unaffected by type 1 diabetes, the mechanism was altered. Cerebral arteries from diabetic rats had an enhanced vasorelaxation response to the precursor of H_2_S, l-cysteine, along with an increase in CSE mRNA. These data show that type 1 diabetes upregulates the endogenous production of H_2_S in the cerebral vasculature. Furthermore, incubating type 1 diabetic cerebral arteries with the H_2_S donor, NaHS, reduces their capacity for superoxide production, thus the upregulation of H_2_S production in diabetes may serve to protect cerebral arteries against oxidative insult.

Vascular dysfunction, of both micro- and macrovessels, is a major cause of morbidity in diabetes. Endothelial dysfunction, in both conductance and resistance arteries, is a primary factor in the pathogenesis of vascular disease in both type 1 and type 2 diabetes (de Vriese et al. 2000; Rask-Madsen and King 2007). Notably, endothelial dysfunction occurs earlier and is of greater severity in cerebral vessels in diabetes (Kitayama et al. 2006). The type 1 model of diabetes used in this study selectively induced cerebral endothelial dysfunction as the response to bradykinin was significantly attenuated, however, smooth muscle cell function was retained. The endothelial dysfunction was accompanied by an increase in cerebral vascular superoxide production. It is well known that an increase in superoxide reduces bioavailability of NO (Gryglewski et al. 1986; Mackenzie and Martin 1998), however, rat cerebral arteries mediate bradykinin-induced vasorelaxation primarily via EDHF (Komatsu et al. 2000), rather than NO. Previous work has shown that diabetes-induced increased oxidative stress also alters endothelial function via changing the contribution of vascular ion channels to vasorelaxation responses (Leo et al. 2010, 2011b,c2011c) and the current observations are in line with this.

Although not extensively studied, acute, exogenous H_2_S (via NaHS) causes cerebral vasodilation via mechanisms involving ion channels including K_ATP_ channels (Leffler et al. 2011; Liang et al. 2011), Ca^2+^ channels (Streeter et al. 2012; Tian et al. 2012), and Cl^−^ channels (Streeter et al. 2012). Although these experiments utilized different techniques: isobaric (Liang et al. 2011), isometric (Streeter et al. 2012; Tian et al. 2012), and in situ cranial windows (Leffler et al. 2011), the EC_50_ value for NaHS-induced vasodilation was similar between the techniques (∼30–100 μmol/L). The question arises as to whether the method used influences the apparent mechanism, as isobaric techniques showed an involvement of K_ATP_ channels (Leffler et al. 2011; Liang et al. 2011), by contrast the isometric techniques clearly show no role of K^+^ channels, or NO, but roles for Ca^2+^ channels (Streeter et al. 2012; Tian et al. 2012) and a DIDS-sensitive component, suggesting an involvement of Cl^−^ channels or Cl^−^/HCO_3_^−^ exchange in the vasorelaxation (Streeter et al. 2012). The mechanism of NaHS-induced vasorelaxation in type 1 diabetes has not been determined previously. The current study shows that the efficacy of the vasorelaxation response is unaltered, but that the DIDS-sensitive component is reduced, suggesting a lesser role for Cl^−^ channels or Cl^−^/HCO_3_^−^ exchange under type 1 diabetic conditions. There is a limitation to this observation, as DIDS has nonspecific activities including inhibiting a variety of Cl^−^ exchangers (Wulff 2008), Na^+^ channels (Liu et al. 1998), ryanodine (Hill and Sitsapesan 2002), and purine (Bultmann and Starke 1994) receptors and K_ATP_ channels (Gojkovic-Bukarica et al. 2002), however, the latter effect cannot infer K_ATP_ channel dependence in this case as the response to NaHS was unaffected by the K_ATP_ channel blocker, glibenclamide (data not shown). Further investigation using more selective Cl^−^ conductance blockers is needed to elucidate this.

While the magnitude of the vasorelaxation response of cerebral arteries to exogenous H_2_S was unaffected by type 1 diabetes, in contrast to this, CSE-dependent vasorelaxation elicited by l-cysteine was significantly enhanced. This suggests that type 1 diabetes upregulates the endogenous production of H_2_S in cerebral arteries. l-cysteine induced a relaxation of cerebral arteries that was attenuated by the CSE inhibitor, PPG, confirming a role for the l-cysteine–CSE–H_2_S pathway, although it is noted that the inhibition by PPG was a shift in sensitivity, rather than a reduction of magnitude. Possible reasons for the incomplete block of l-cysteine-induced vasorelaxation by PPG are (i) only a proportion of the vasorelaxation induced by l-cysteine was due to H_2_S production, (ii) l-cysteine was being converted to H_2_S via an alternate enzyme, for example, 3-MST (the presence of which has been demonstrated in vascular tissues [Shibuya et al. 2009; ]), or (iii) there was incomplete inhibition of CSE by PPG. The latter could be due to the relatively poor cell permeability of PPG (Marcotte and Walsh 1976). It should also be noted that PPG acts by covalently binding to the pyridoxal 5′-phosphate (PLP) (cofactor) binding site of the CSE enzyme and thus may also influence other PLP-dependent enzymes (Thompson et al. 1982). Despite these limitations, PPG is a widely used inhibitor of endogenous H_2_S production and is the best available pharmacological tool to inhibit CSE at this time.

The enhanced vasorelaxant efficacy of l-cysteine in type 1 diabetic cerebral arteries suggests enhanced generation of H_2_S via either enhanced activity or expression of CSE. Indeed, there was also increased CSE mRNA expression in type 1 diabetic compared to control cerebral arteries. Previous observations suggest that type 1 diabetes may indeed upregulate vascular CSE expression, in the aorta of NOD mice as CSE mRNA and protein expression were enhanced in a manner that correlated with disease severity (Brancaleone et al. 2008). In another study, however, CSE mRNA and protein expression in aorta of type 1 diabetic rats were not significantly different from control, as determined by RT-PCR and western blotting (Denizalti et al. 2011). Although little is known about the regulation of CSE activity, there is some evidence that CSE function is redox regulated (Hassan et al. 2012). Diabetes-induced vascular ROS generation may result in upregulation of CSE activity and the results of the current study support this idea. On the other hand the cerebral artery endothelial function is not totally protected by an upregulation of CSE, and this may be due to the vast oxidative insult of this type 1 diabetes model, but this will require further investigation.

H_2_S has been reported to act as a scavenger for a variety of ROS, including superoxide (Yan et al. 2006), hydrogen peroxide (Muzaffar et al. 2008b), peroxynitrite (Whiteman et al. 2004), and hypochloride (Whiteman et al. 2005). It has previously been shown that H_2_S can inhibit NADPH oxidase in cell-based studies (Muzaffar et al. 2008a,b) and additionally that NaHS can reverse the upregulation of several NADPH oxidase subunits observed in the thoracic aorta of diabetic rats (Zheng et al. 2010). It has been suggested that upregulated H_2_S production in diabetes may thus form part of a protective mechanism against excessive ROS generation (Yusuf et al. 2005; Kaneko et al. 2009; Hassan et al. 2012). The effects of exogenous H_2_S on vascular NADPH-stimulated superoxide generation were therefore assessed in type 1 diabetic compared to control cerebral arteries. Type 1 diabetes enhanced NADPH-stimulated superoxide generation in cerebral arteries, indicating that NADPH oxidase expression or activity is upregulated by type 1 diabetes in the vasculature, in line with the literature (Shen 2010). Prior incubation of vessels in the H_2_S donor, NaHS, attenuated superoxide production in diabetic cerebral arteries back to control levels, but did not influence superoxide production in control cerebral arteries, indicating that H_2_S can selectively normalize superoxide production in cerebral arteries from type 1 diabetic animals. The design of the experiment was such that the effects of H_2_S could not be due to its direct scavenging effect, as it was removed before measuring NADPH-stimulated superoxide production, suggesting that H_2_S acted by inhibition of superoxide-generating enzymes, such as NADPH oxidase.

In this study, NaHS was used to deliver H_2_S. This salt is commonly used for this purpose and is considered a “fast” releaser of NaHS, rapidly increasing H_2_S concentrations (Li et al. 2008). In addition H_2_S is highly volatile and diffuses rapidly (Deleon et al. 2012) so the final concentration under the experimental conditions is likely to be lower than that stated, indeed previous work suggests this may be at least 10-fold (Al-Magableh and Hart 2011). The physiological concentration of H_2_S is difficult to measure, and although previously reported in the μmol/L range it is now considered to be in the nM range (Furne et al. 2008), thus the concentrations used in this study are likely to be physiologically relevant.

The findings of this and other studies (Yusuf et al. 2005; Kaneko et al. 2009) suggest that H_2_S may form part of an important adaptive response to oxidative stress in type 1 diabetes. Given the vasodilator action of H_2_S, such an adaptive response could also counteract the enhanced level of tone in diabetic cerebral vessels associated with reduced NO bioavailability and enhanced myogenic reactivity. However, more research will be required to confirm whether the CSE–H_2_S pathway forms part of a regulatory response to diabetic oxidative stress, and whether manipulation of this system can be applied to therapeutics.

The key findings of the present study were that type 1 diabetes increased the vasorelaxant efficacy of endogenous H_2_S in rat cerebral arteries and enhanced vascular biosynthesis of H_2_S, as assessed by increased vasorelaxation to the H_2_S substrate l-cysteine and increased CSE mRNA production in the type 1 diabetic group. Vasorelaxation mediated by exogenous H_2_S was retained under diabetic conditions, although the molecular mechanism was altered. A selective attenuation of pathologically increased superoxide by exogenous H_2_S was also observed in diabetic cerebral arteries. The study shows the CSE–H_2_S pathway is altered in diabetes and that endogenous H_2_S may counter the vascular oxidative stress apparent in this condition.
